# Relationships between Obesity and Incidence of Fractures in a Middle‐Aged Population: A Study from the CARTaGENE Cohort

**DOI:** 10.1002/jbm4.10730

**Published:** 2023-03-03

**Authors:** Anne‐Frédérique Turcotte, Sonia Jean, Suzanne N. Morin, Fabrice Mac‐Way, Claudia Gagnon

**Affiliations:** ^1^ Endocrinology and Nephrology Unit CHU de Quebec‐Université Laval Research Centre Quebec City Quebec Canada; ^2^ Quebec Heart and Lung Institute Research Centre Quebec City Quebec Canada; ^3^ Faculty and Department of Medicine Laval University Quebec City Quebec Canada; ^4^ Bureau d'information et études en santé des populations Institut national de santé publique du Québec Québec City Quebec Canada; ^5^ Department of Social and Preventive Medicine Laval University Quebec City Quebec Canada; ^6^ Department of Medicine, Faculty of Medicine McGill University Montreal Quebec Canada

**Keywords:** ABDOMINAL OBESITY, EPIDEMIOLOGY, FRACTURE RISK, OBESITY

## Abstract

The association between obesity and fracture risk is complex and may vary by definition of obesity, skeletal site, and sex. We aimed to evaluate the relationships between obesity, defined using body mass index (BMI) or waist circumference (WC), and fracture incidence at any site and by skeletal site (i.e., major osteoporotic fractures [MOFs], distal lower limb fractures [tibia, ankle, feet], and distal upper limb fractures [forearm/elbow, wrist]). The secondary aim was to assess the aforementioned relationships by sex. We used CARTaGENE, a large population‐based cohort of individuals aged 40–70 years from Quebec, Canada, who were assessed in 2009–2010. Incident fractures were identified via linkage with healthcare administrative databases over a 7‐year period. Cox proportional hazard models adjusted for several potential confounders were used to estimate the relationships, with exposures treated as continuous variables. Results are reported as adjusted hazard ratios (aHRs) and 95% confidence intervals. We identified 19 357 individuals (mean ± standard deviation: age 54 ± 8 years, BMI 27 ± 5 kg/m^2^, WC 94 ± 14 cm; 51.6% women). During follow‐up, 497 women and 323 men sustained a fracture. There was a linear relationship between fracture incidence and WC, while cubic splines best fitted the relationship for BMI. Greater WC was associated with an increased risk of fracture at the distal lower limbs in the whole cohort and in the subgroup of women: aHR for each 10 cm increased in WC of 1.12 (1.03, 1.21) and 1.12 (1.01, 1.24), respectively. In men, WC was not significantly associated with any fracture outcome. Higher BMI was also significantly associated with distal lower limb fracture risk in the whole cohort (*p* = 0.018). No significant relationships were found between either WC or BMI and the risk of any fracture, MOFs, and distal upper limb fractures. In middle‐aged individuals, obesity, and mainly abdominal obesity, was associated with distal lower limb fracture risk. © 2023 The Authors. *JBMR Plus* published by Wiley Periodicals LLC on behalf of American Society for Bone and Mineral Research.

## Introduction

More than 50% of adults worldwide are overweight or obese, with no sign that the increased prevalence observed over recent decades is abating.^(^ ^1^, ^2^ ^)^ Obesity has long been thought to be a protective factor against fractures. However, the relationship between obesity and fracture risk is complex, and recent literature suggests that it may vary depending on the definition of obesity. While most studies found that a higher body mass index (BMI), a measure of general obesity, was associated with a reduced incidence of hip fracture,^(^ ^3^, ^4^, ^5^ ^)^ a positive association between waist circumference (WC), an indicator of abdominal obesity, and hip fracture risk was reported in a recent meta‐analysis.^(^ ^6^ ^)^ In addition, studies suggest that obesity predisposes a person to fracture at certain skeletal sites, including the ankle, upper leg, and proximal humerus.^(^ ^7^, ^8^, ^9^ ^)^ Abdominal obesity is recognized as a stronger risk factor for metabolic disorders than BMI,^(^ ^10^ ^)^ which may also hold true for bone fragility and fracture risk. Indeed, abdominal obesity is associated with greater insulin resistance, oxidative stress, increased circulating inflammatory cytokines, and altered levels of bone‐regulating hormones,^(^ ^11^, ^12^, ^13^ ^)^ all of which are known to adversely affect bone metabolism. Moreover, BMI is a less accurate measure of adiposity in older adults due to change in body composition associated with aging.^(^ ^14^, ^15^ ^)^

We recently conducted a systematic review and meta‐analysis looking at the association between obesity and fracture risk and found several knowledge gaps in the literature.^(^ ^9^ ^)^ First, the majority of the studies used BMI to define obesity, and among the limited number of studies that used WC, only the associations with hip and vertebral fracture risk were assessed.^(^ ^16^, ^17^, ^18^, ^19^, ^20^ ^)^ Moreover, all the studies used the BMI international classification categories from the World Health Organization (WHO).^(^ ^21^ ^)^ Although this classification has been shown to predict cardiometabolic events, it is uncertain whether this is the case for fractures. In addition, studies often combined the overweight and obesity categories, which may add bias to the results. Moreover, no study has yet determined the linear or nonlinear relationship between BMI or WC and fracture risk, which is clinically relevant to identifying adequately individuals at risk of fracture. Finally, even though there are major differences in bone metabolism and fracture risk between men and women,^(^ ^22^, ^23^ ^)^ numerous studies analyzed men and women together. This may hide a potential sex‐specific relationship between obesity and fracture risk.

We thus aimed to evaluate the relationships between obesity (defined by BMI or WC) and fracture incidence at any site and at specific skeletal sites using a large population‐based cohort representative of the Province of Quebec (Canada). A secondary aim was to evaluate those relationships by sex.

## Materials and Methods

### Study design and population

We used CARTaGENE, a large population‐based cohort of community‐dwelling individuals aged 40–70 years from the province of Quebec, Canada.^(^ ^24^ ^)^ The CARTaGENE cohort originally aimed at evaluating the associations between genotypes and the prevalence of chronic diseases. Briefly, participants were selected randomly between 2009 and 2010 using provincial health insurance registries to be representative of the Quebec metropolitan population (Montreal, Quebec City, Sherbrooke, Saguenay). Individuals were not eligible for inclusion if they resided outside of the selected regions, were living in First Nations Reserves, long‐term health care facilities, or correctional facilities. For this study, all individuals with available BMI and WC measurements were included. This study was approved by the CHU de Québec‐Université Laval Ethics Committee and was conducted in accordance with the ethical statement of the Declaration of Helsinki.

### Data sources

Participants were evaluated at a single baseline visit, during which they completed questionnaires, underwent anthropometric measurements, and provided blood samples. Sociodemographic and lifestyle questionnaires were self‐administered, but the health questionnaire was administered by a trained interviewer. All participants signed the informed consent form before joining the study. They also agreed to be followed through linkage with the Quebec healthcare administrative databases, including the medical service and drug claims databases, and the hospital discharge database.

### Fracture incidence

Incident fractures from recruitment (2009–2010) to the end of study follow‐up (March 31, 2016) were identified through record linkage with the Quebec healthcare administrative databases using a previously validated algorithm.^(^ ^25^ ^)^ Briefly, an incident fracture was confirmed through medical service codes for fracture treatment (open reduction, closed reduction, and immobilization) or a visit with an orthopedic surgeon, combined with any other claim associated with a fracture diagnosis. The algorithm has a sensitivity and a positive predictive value of at least 80% for each nonvertebral skeletal site.^(^ ^25^ ^)^ Any fracture site was considered, apart from craniofacial, toe, and digit fractures. The outcomes considered were any fracture, major osteoporotic fractures (MOFs) (i.e., hip, pelvis, femur, vertebrae, humerus, wrist), distal lower limb fractures (tibia, ankle, foot), and distal upper limb fractures (wrist, forearm/elbow). Time from recruitment to the first fracture event was obtained using the recruitment date and the date of the first code associated with a fracture in the Quebec healthcare administrative databases. Individuals were censored at the time of fracture, death, if they became nonadmissible to the Quebec healthcare insurance plan during follow‐up, or at the end of study follow‐up.

### Obesity and confounders assessment

Exposures of interest (obesity) were BMI and WC at the recruitment visit. Height and weight were measured, and BMI was calculated as weight (kg) divided by squared height (m^2^). Waist circumference was measured three times using a measuring tape and following standard protocol.^(^ ^24^ ^)^ The mean of the three WC measurements was calculated, and BMI and WC were used as continuous variables.

Information on sociodemographic factors, calcium and vitamin D intake from supplements, comorbidities, and medication were collected from the questionnaires completed at baseline. Prevalent type 2 diabetes cases were confirmed if they met one of the following criteria: (1) self‐reported type 2 diabetes diagnosis combined with use of oral hypoglycemic medication or insulin, (2) self‐reported type 2 diabetes diagnosis combined with a glycated hemoglobin (HbA_1c_) ≥6.5% or random glucose ≥11.1 mmol/L, (3) use of oral hypoglycemic agents or insulin, or (4) HbA_1c_ ≥6.5% and random glucose ≥11.1 mmol/L. Fracture history in the past 5 years was obtained from the Quebec healthcare administrative databases using the same algorithm as described earlier. Bone parameters (i.e., speed of sound, broadband ultrasound attenuation, stiffness index, and *t*‐score) were derived from quantitative ultrasound (QUS; Lunar Achilles Express, GE Healthcare, Madison, WI, USA).

### Statistical analyses

Descriptive analyses were performed separately for individuals who sustained or did not sustain a fracture during follow‐up. Student's *t* tests were used for comparisons of continuous variables and chi‐squared tests for categorical variables. Relationships between obesity and fracture incidence were assessed using the Cox proportional hazards model, with exposure of interest (BMI or WC) as a continuous variable and time to fracture as the outcome. Results were reported as hazard ratios (HRs) with corresponding 95% confidence intervals (CIs). Models were performed for the entire cohort (primary aim) and for men and women separately (secondary aim). Person‐time at risk was calculated from the date of recruitment, in days, to the first event among the following: first fracture, death, end of admissibility to the Quebec healthcare insurance plan during follow‐up, or end of study follow‐up (March 31, 2016). For each fracture outcome we used the Cox proportional hazard models to perform univariate and multivariate models adjusted for all potential confounding factors. Potential confounders were identified using a directed acyclic graph (DAG), presented in Figure S1 in Appendix S1, which was used to identify the minimally sufficient adjustment sets of variables, intermediate variables, and colliders.^(^ ^26^ ^)^ The causal relationships between variables were established a priori, based on the literature. Therefore, intermediate factors were not conditioned on in the analyses. For each model, BMI and WC were considered in four parametric forms: linear and cubic splines with 0, 3, and 4 knots. The best model was selected as the model with the smallest Akaike information criterion (AIC) and Bayesian information criterion (BIC). Regression line cut points (i.e., the WC or BMI value associated with a null risk of fracture) were determined with a linear regression, in which WC and BMI were used as outcomes and fracture risk as the exposure. These linear regression models were adjusted for all covariates identified in the DAG.

To evaluate the robustness of our results, we conducted the following sensitivity analyses: (1) we used the Fine‐Gray model, accounting for the competing risk of mortality, for each exposure of interest and fracture outcome; (2) since menopause and type 2 diabetes are both associated with an increased risk of fracture, we tested the modifying effect of menopausal status and type 2 diabetes on the relationships between BMI or WC and all fracture outcomes by adding an interaction term in the fully adjusted Cox proportional hazards models; (3) we excluded individuals with osteoporosis or taking osteoporosis medication to test whether the adjustment for these factors was sufficient; (4) we analyzed humerus as a specific fracture site since results on its association with obesity are conflicting among studies; (5) we analyzed specifically ankle fractures since previous studies showed a strong association between BMI and this fracture site; and (6) we excluded individuals aged 40–49 years since the mechanism of fracture is potentially different for individuals in this age group, i.e., higher likelihood of traumatic fractures. All analyses were conducted using SAS software version 9.4 (SAS Institute, Cary, NC, USA). All tests were two‐sided, and a significant level of 0.05 was used for all analyses.

## Results

### Population characteristics

The baseline characteristics of the individuals with and without an incident fracture are presented in Table 1. From the 19 990 individuals initially recruited in CARTaGENE, 19 357 (9985 women and 9372 men) had measurements of WC and 18 654 (9743 women and 8911 men) had BMI assessed at recruitment. During the 7‐year follow‐up period (median time of 5.8 years), 820 individuals sustained a fracture: 497 women (260 MOFs, 219 distal lower limb, and 141 distal upper limb fractures) and 323 men (155 MOFs, 134 distal lower limb, and 62 distal upper limb fractures). Compared with those without a fracture, individuals who sustained an incident fracture were older, more likely to be menopausal women, and to be living alone and had a lower education level and annual income and more comorbidities. They were also more likely to have a history of fracture, to take calcium and vitamin D supplements, and to use medications. Missing data for exposure variables were modest: BMI (6.7%) and WC (3.2%). Data for potential confounders were complete except for annual income (6.1%), education level (0.6%), marital status (0.6%), alcohol consumption (0.7%), and physical activity (4.6%).

**Table 1:** Baseline Characteristics of CARTaGENE Cohort by Fracture Incidence (*n* = 19 357)

Variables	No fracture (*n* = 18 537)	Incident fracture (*n* = 820)	*p* value*
Sociodemographic characteristics			
Age (years)	54 ± 8	56 ± 8	<0.001
40–49	6557 (35.4)	204 (24.9)	
50–59	7005 (37.8)	362 (44.2)	
60–69	4975 (26.8)	254 (31.0)	
≥65	2278 (12.3)	120 (14.6)	
Women	9488 (51.2)	497 (60.6)	<0.001
Menopausal women	4484 (47.3)	304 (61.2)	<0.001
Ethnicity			
European ancestry	16 555 (89.3)	776 (94.6)	<0.001
Latino	409 (2.2)	13 (1.6)	
African	382 (2.1)	7 (0.9)	
Asian	421 (2.3)	8 (1.0)	
Other	770 (4.2)	16 (1.9)	
Marital status			
Married	11 863 (64.0)	462 (56.3)	<0.001
Single/divorced/separated/widowed	6 573 (35.5)	351 (42.8)	
Missing	101 (0.5)	7 (0.9)	
Education level			
≤ High school	4 759 (25.7)	229 (27.9)	0.039
> High school	13 676 (73.8)	582 (71.0)	
Missing	102 (0.6)	9 (1.1)	
Annual household income			
< $25 000	2 253 (12.2)	148 (18.1)	<0.001
≥ $25 000	15 159 (81.8)	622 (75.8)	
Missing	1 125 (6.1)	50 (6.1)	
Lifestyle habits and clinical parameters			
Body mass index (kg/m^2^)	27 ± 5	27 ± 5	0.904
Waist circumference (cm)	94 ± 14	94 ± 15	0.706
BMD *t*‐score (QUS)	0.206 ± 1.186	−0.257 ± 1.093	<0.001
Type 2 diabetes			
Insulin treated	238 (1.3)	28 (3.4)	<0.001
Treated with oral antidiabetic agents	1 560 (8.4)	60 (7.3)	
No type 2 diabetes	16 739 (90.3)	732 (89.3)	
Active smoking	2 660 (14.4)	137 (16.7)	0.060
Alcohol consumption (servings/week)	5 ± 9	5 ± 10	0.184
Physical activity level (METS × hours per week)	51 ± 48	50 ± 49	0.356
History of fracture			
Within the last year	110 (0.6)	20 (2.4)	<0.001
Within the last 5 years	586 (3.2)	84 (10.2)	<0.001
Supplementary calcium intake	3 794 (20.5)	223 (27.2)	<0.001
Supplementary vitamin D intake	3 665 (19.8)	215 (26.2)	0.001
Diseases			
Osteoporosis	1 398 (7.5)	120 (14.6)	<0.001
Arthritis	853 (4.6)	64 (7.8)	<0.001
Hyperthyroidism	1 307 (7.1)	65 (7.9)	0.339
Type 1 diabetes	106 (0.6)	14 (1.7)	<0.001
Liver disease	271 (1.5)	21 (2.6)	0.012
Chronic obstructive pulmonary disease	1 060 (5.7)	71 (8.7)	<0.001
Cardiovascular disease	1 197 (6.5)	53 (6.5)	0.995
Cancer	1 478 (8.0)	80 (9.8)	0.066
Medications			
Osteoporosis drugs	636 (3.4)	66 (8.1)	<0.001
Glucocorticoids	148 (0.8)	6 (0.7)	0.833
Anticonvulsants	564 (3.0)	46 (5.6)	<0.001

Data are presented as mean ± SD or *n* (%).

BMD = bone mineral density; QUS = quantitative ultrasound; METS = metabolic equivalent of task.

* Student's *t* test or chi‐squared test between groups.

### Relationships and associations between WC and fracture risk

**Fig. 1:**
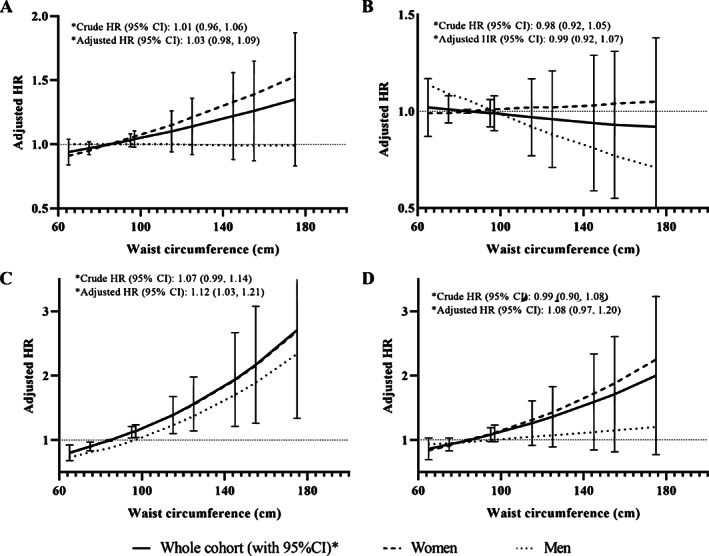
Linear relationships between waist circumference and incidence of (*A*) any fractures, (*B*) major osteoporotic fractures, (*C*) distal lower limb fractures, and (*D*) distal upper limb fractures, in the whole cohort and in men and women separately. Hazard ratios are per 10‐cm increase in waist circumference. HR: hazard ratio; 95% CI: 95% confidence interval. Models are adjusted for age, sex, menopausal status, area of residence, ethnicity, education, annual income, marital status, smoking, alcohol intake, physical activity level, calcium and vitamin D supplementation, history of fracture, osteoporosis, hyperthyroidism, chronic kidney disease, type 1 diabetes, liver disease, chronic obstructive pulmonary disease, cardiovascular disease, cancer, osteoporosis drugs, glucocorticoids, anticonvulsants, oral contraceptive, hormonal replacement therapy, proton pump inhibitor, and blood pressure medication.

For all models with WC as the exposure of interest, the analysis showed that the best fit was with the linear model. The cut points on the regression lines associated with a null risk of fracture were 85 cm for the whole cohort, 84 cm in women, and 97 cm in men. Results of the unadjusted and adjusted linear relationships between WC and fracture outcomes are presented in Table 2. Figure 1*A*–*D* illustrates the adjusted linear relationships between WC and fracture outcomes for the entire cohort and for men and women separately. In models including the entire cohort, a greater WC was significantly and linearly associated with an increased risk of fracture only at the distal lower limbs. Adjusted HR for each 10‐cm increase in WC was 1.12 (95% CI: 1.03, 1.21). Similarly, a higher WC was significantly associated with an increased risk of distal lower limb fractures in women (HR per 10 cm increase: 1.12 [95% CI: 1.01, 1.24]). In men, WC was not significantly associated with the risk of distal lower limb fracture. Moreover, no significant relationships between WC and the risk of any fracture, MOFs, and distal upper limb fractures were observed in any of the groups. Table S1 in Appendix S1 presents the HR (95% CI) of all fracture outcomes by category of WC for the whole cohort.

**Table 2:** Linear Relationships Between Waist Circumference and Fracture Outcomes in Whole Cohort and in Men and Women Separately

Population	Fracture outcome	Waist circumference	Incident fracture, *n*	Crude HR (95% CI)	*p* value	Adjusted HR (95% CI)^a^	*p* value
Whole cohort	Any fracture	HR per 10‐cm increase	820	1.01 (0.96, 1.06)	0.700	1.03 (0.98, 1.09)	0.225
	MOF	HR per 10‐cm increase	415	0.98 (0.92, 1.05)	0.524	0.99 (0.92, 1.07)	0.804
	Distal lower limb	HR per 10‐cm increase	353	1.07 (0.99, 1.14)	0.073	1.12 (1.03, 1.21)	0.006
	Distal upper limb	HR per 10‐cm increase	203	0.99 (0.90, 1.08)	0.760	1.08 (0.97, 1.20)	0.157
Women	Any fracture	HR per 10‐cm increase	497	1.10 (1.03, 1.18)	0.003	1.05 (0.98, 1.12)	0.162
	MOF	HR per 10‐cm increase	260	1.06 (0.98, 1.16)	0.124	1.01 (0.92, 1.10)	0.915
	Distal lower limb	HR per 10‐cm increase	219	1.15 (1.05, 1.25)	0.002	1.12 (1.01, 1.24)	0.026
	Distal upper limb	HR per 10‐cm increase	141	1.11 (0.99, 1.24)	0.062	1.09 (0.97, 1.24)	0.157
Men	Any fracture	HR per 10‐cm increase	323	1.00 (0.92, 1.09)	0.901	1.00 (0.91, 1.10)	0.975
	MOF	HR per 10‐cm increase	155	0.99 (0.87, 1.12)	0.831	0.96 (0.84, 1.10)	0.535
	Distal lower limb	HR per 10‐cm increase	134	1.11 (0.98, 1.26)	0.091	1.11 (0.97, 1.27)	0.123
	Distal upper limb	HR per 10‐cm increase	62	1.02 (0.85, 1.23)	0.850	1.02 (0.83, 1.26)	0.823

^a^ Model adjusted for age, sex, menopausal status, area of residence, ethnicity, education, annual income, marital status, smoking, alcohol intake, physical activity level, calcium and vitamin D supplementation, history of fracture, osteoporosis, hyperthyroidism, chronic kidney disease, type 1 diabetes, liver disease, chronic obstructive pulmonary disease, cardiovascular disease, cancer, osteoporosis drugs, glucocorticoids, anticonvulsants, oral contraceptive, hormonal replacement therapy, proton pump inhibitor, blood pressure medication.

### Relationships and associations between BMI and fracture risk

**Fig. 2:**
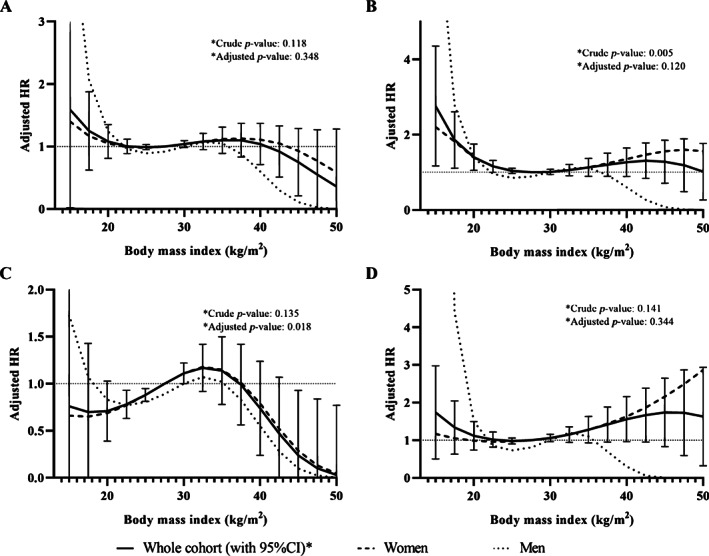
Nonlinear relationships between body mass index and incidence of (*A*) any fractures, (*B*) major osteoporotic fractures, (*C*) distal lower limb fractures, and (*D*) distal upper limb fractures, in the whole cohort and in men and women separately. HR: hazard ratio; 95% CI: 95% confidence interval. Models are adjusted for age, sex, menopausal status, area of residence, ethnicity, education, annual income, marital status, smoking, alcohol intake, physical activity level, calcium and vitamin D supplementation, history of fracture, osteoporosis, hyperthyroidism, chronic kidney disease, type 1 diabetes, liver disease, chronic obstructive pulmonary disease, cardiovascular disease, cancer, osteoporosis drugs, glucocorticoids, anticonvulsants, oral contraceptive, hormonal replacement therapy, proton pump inhibitor, and blood pressure medication.

For models with BMI as the exposure of interest, the analysis showed that the best model fit was obtained using cubic splines without knots. BMI was treated flexibly by comparing each multiple of 2.5, from 15 to 50 kg/m^2^, to the reference value. The reference value was 28 kg/m^2^ for the whole cohort, 27 kg/m^2^ for models including women only, and 30 kg/m^2^ for models including men (Fig. 2*A*–*D*). Table 3 shows results of the relationships between BMI and fracture outcomes. In models including the whole cohort, BMI was significantly associated with the risk of distal lower limb fractures in the fully adjusted model (*p* = 0.018), which was also close to reach significance in women (*p* = 0.056), but not in men (*p* = 0.504). Compared with the BMI reference value of 28 kg/m^2^, the risk of fracture at the distal lower limbs decreased linearly from 22% to 12% in individuals with a BMI between 25 and 22.5 kg/m^2^ and increased by 11% in individuals with a BMI of 30 kg/m^2^ (Fig. 2*C*). A similar relationship was observed in women. No other significant relationship between BMI and the other fracture outcomes was observed in the whole cohort or in women. In men, a significant relationship was observed between BMI and distal upper limb fractures (*p* = 0.049), but the results were influenced by the extreme low BMI values (Fig. 2*D*). Table S2 in Appendix S1 presents the HR (95% CI) of all fracture outcomes by category of BMI for the whole cohort.

**Table 3:** Nonlinear relationships between body mass index and fracture outcomes in whole cohort and in men and women separately

Population	Fracture outcome	Incident fracture, *n*	Crude *p* value for spline	Adjusted^a^ *p* value for spline
Whole cohort	Any fracture	776	0.118	0.348
	MOF	392	0.005	0.120
	Distal lower limb	336	0.135	0.018
	Distal upper limb	202	0.141	0.344
Women	Any fracture	477	0.371	0.688
	MOF	254	0.251	0.185
	Distal lower limb	209	0.065	0.056
	Distal upper limb	139	0.325	0.192
Men	Any fracture	299	0.016	0.111
	MOF	138	0.008	0.136
	Distal lower limb	127	0.337	0.504
	Distal upper limb	63	0.011	0.049

^a^ Model adjusted for age, sex, menopausal status, area of residence, ethnicity, education, annual income, marital status, smoking, alcohol intake, physical activity level, calcium and vitamin D supplementation, history of fracture, osteoporosis, hyperthyroidism, chronic kidney disease, type 1 diabetes, liver disease, chronic obstructive pulmonary disease, cardiovascular disease, cancer, osteoporosis drugs, glucocorticoids, anticonvulsants, oral contraceptive, hormonal replacement therapy, proton pump inhibitor, blood pressure medication.

### Sensitivity analyses

As shown in Table S3 in Appendix S1 accounting for the competing risk of mortality did not alter our results for all models using WC but further strengthened the associations. However, for models using BMI in men, the associations between BMI and both any fracture and MOF outcomes became significant (*p* = 0.040 and *p* = 0.042, respectively). As seen previously, only men with a BMI of ≤17.5 kg/m^2^ had a significantly higher risk of fracture than men with a BMI of 30 kg/m^2^. Similarly, excluding individuals with osteoporosis or taking osteoporosis medication did not change our findings but strengthened the associations (Table S3 in Appendix S1). Although we found no significant relationship between humerus fracture risk and either WC or BMI, significant linear relationships between WC and ankle fracture risk were observed in the whole cohort, as well as in men and women (adjusted HR for each 10‐cm increase in WC: 1.21 [95% CI: 1.07, 1.37]; 1.25 [95% CI: 1.01, 1.56]; and 1.19 [95% CI: 1.02, 1.39], respectively). When BMI was used as the exposure, the relationship was only significant in the whole cohort (*p* = 0.026) and was similar to what was observed for distal lower limb fractures (Figure S2 *A*,*B*, Tables S4, S5 in Appendix S1). Interaction terms for both type 2 diabetes and menopausal status were not significant in any of the models. Finally, excluding individuals aged 40–49 also strengthened our results in models with WC as the exposure. In fact, linear relationships between WC and fracture incidence were a bit stronger, especially with regards to distal lower limb fractures (adjusted HR for each 10‐cm increase in WC: 1.16 [95% CI: 1.06, 1.28]). Relationships between BMI and fracture outcomes were unchanged (Table S3 in Appendix S1).

## Discussion

Our study brings novel insights into the intricate relationship between obesity and fracture risk. In this community‐based cohort of 19 357 individuals aged 40–70 years followed for up to 7 years, the relationships between obesity and fracture risk clearly differed whether WC or BMI was used to define obesity. Moreover, obesity was a risk factor for only distal lower limb fractures in this age group, which seemed mainly driven by ankle fractures. While relationships were similar between sexes for WC, that was not the case for BMI.

Our study is the first to determine the relationships between WC or BMI and fracture risk at any site and by skeletal site. It revealed that WC is following a linear relationship with fracture risk, being significant for distal lower limb fractures. Although this association reached significance only in the whole cohort and in women, the relationship was similar for men. With regard to BMI, the relationship with distal lower limb fractures in the whole cohort and in women was mostly linear for BMIs ranging between 20 and 32.5 kg/m^2^, resembling the linear relationship observed with WC. In men, no association was observed. The relationships between BMI and fracture risk at any site, MOFs and distal upper limbs seemed to follow a U‐shaped curve. However, spline models were not significant and were influenced by the limited number of fracture events in individuals within the extremes of BMI values (<20 kg/m^2^ and >40 kg/m^2^). The robustness of our results is increased by making adjustments for several potential confounders and the conduct of sensitivity analyses. The fact that relationships between WC and fracture incidence were stronger after exclusion of individuals with osteoporosis or those aged 40–49 years is in line with our hypothesis that visceral adiposity may directly impair bone health and induce bone fragility. Indeed, osteoporosis is associated with an increased risk of fragility fractures and adults aged 40–49 years are more susceptible to fractures caused in a context of workplace or sport injury. Exclusion of these individuals reduced biases by selecting a more homogeneous group of individuals.

Our findings are in line with previous studies that reported an increased risk of ankle fractures in postmenopausal women with BMI‐defined obesity compared with normal‐weight women.^(^ ^7^, ^27^ ^)^ Our study extends these findings to a younger cohort that includes premenopausal women and younger men. It also reveals that a linear relationship exists between distal lower limb fracture risk and WC. To our knowledge, only hip and vertebral fracture sites have been assessed in relation to WC or another marker of abdominal obesity.^(^ ^16^, ^17^, ^18^, ^19^, ^20^ ^)^ Unfortunately, we were not able to confirm whether WC was associated with hip and vertebral fracture risk since the number of fracture events at these skeletal sites was too small. This is likely due to the relatively young age of our study population and the underestimation of vertebral fractures in the healthcare administrative databases. Nonetheless, our study is the first to reveal a linear relationship between WC and distal lower limb fracture risk in middle‐aged men and women. It has clinical implications and reinforces the importance of considering WC along with BMI when screening for individuals at risk of fractures.

In our study, BMI and, perhaps more so, WC were strongly associated with the risk of distal lower limb and ankle fractures. From a mechanical perspective, ankle fractures usually occur following rotation of the talus within the mortise joint. Twisting of the ankle may cause higher torques on bones and lead to fracture in individuals with obesity.^(^ ^28^ ^)^ In addition, most fractures result from a fall,^(^ ^29^ ^)^ and individuals with obesity fall 10%–50% more frequently than individuals with a normal weight.^(^ ^30^, ^31^ ^)^ Fall susceptibility in connection with obesity may be explained by muscle weakness as well as impaired mobility, physical function, and balance.^(^ ^32^, ^33^, ^34^, ^35^ ^)^ Moreover, ankles and distal tibias are not protected with overlying soft tissue padding and must support greater body weight during a fall, perhaps explaining the increased fracture propensity for these sites. It has also been proposed that the risk of falling may be more affected by abdominal obesity, but results are conflicting. A study revealed that android obesity was associated with greater risk of falls,^(^ ^36^ ^)^ while another study found opposite results.^(^ ^37^ ^)^ In addition, abdominal obesity was positively associated with severe falls requiring medical care and resulting in fractures.^(^ ^38^ ^)^ A potential mechanism is that increased body weight has a significant impact on the absolute ankle muscular torque needed to stabilize the body.^(^ ^39^ ^)^ Moreover, individuals with obesity have a delayed motor response, characterized by greater torque onset and time to peak torque, when the body is submitted to postural perturbations. This effect is even more pronounced when an anterior position of the center of mass is present, suggesting that individuals with abdominal obesity may be at higher risk of falling.^(^ ^39^ ^)^ From a metabolic perspective, studies have found that visceral fat is negatively associated with BMD and bone strength^(^ ^40^, ^41^, ^42^ ^)^ and positively associated with nonvertebral fracture risk in women without obesity,^(^ ^43^ ^)^ suggesting that visceral fat adversely affects bone health. In any case, future research is necessary to investigate the mechanisms underlying the higher risk of distal lower limb fractures, and especially ankle fractures, in people with obesity and abdominal obesity.

As opposed to what was reported in numerous studies, our study failed to detect associations between increased BMI and a reduced risk of fracture at certain skeletal sites, namely the hip and upper limbs. This suggests that individuals with obesity, at least in this age group, have a similar fracture risk than overweight and even normal weight individuals at some skeletal sites. These findings are in line with a study that found a similar prevalence of nonvertebral low‐trauma fractures in women with versus without obesity aged 40 years and older from São Paolo, Brazil, regardless of BMD and other clinical risk factors.^(^ ^44^ ^)^ This is in contrast to the widespread belief in a protective effect of obesity in fractures. Our results, however, need to be interpreted with caution given the small number of hip fractures in our cohort. A possible explanation for divergent results between our study and previous studies is the fact that BMI was not measured in several studies but self‐reported or retrieved from patient files, being thus exposed to misclassification bias. Moreover, most studies treated BMI in categories and sometimes mixed overweight and obese individuals together when compared to normal‐weight individuals. Nevertheless, our results may suggest that the protective effect of obesity in fractures at certain skeletal sites is mostly true in the elderly, who are more susceptible to bone fragility.

Another interesting finding from our study concerns the cut points of the relationships. First, we found that the WHO categorization for WC was accurate in identifying individuals at risk of fractures. Indeed, the cut points (i.e., the value associated with a null risk) of the regression lines were 84 and 97 cm in women and men, respectively, which is within the range of values associated with an increased risk of cardiometabolic complications (80–88 cm for women and 94–102 cm for men). However, we found that the relationships between WC and fracture incidence were linear, so using WC categories to evaluate fracture risk may be biased through over‐ or underestimation of certain individuals grouped in the same category. On the other hand, we found that the BMI international classification was not following the relationship with fracture incidence. Therefore, classifying individuals using the WHO BMI categorization does not discriminate individuals who are at a higher or a lower risk of fracture. It creates heterogeneity within categories and, thus, affects the validity of the results.

The strengths of this study include the prospective design and the large community‐based sample, which was not selected for adiposity‐related traits. Moreover, the age group studied had not been studied thoroughly previously. In addition, the distributions of sociodemographic characteristics in the CARTaGENE cohort were similar to the distribution in the general population.^(^ ^24^ ^)^ Moreover, assessment of multiple clinical characteristics was performed following rigorous methods, which allowed adjustment for various potential confounders and limited the number of missing data. Anthropometric measures were also assessed following standard protocols, and fractures were identified through a validated algorithm, reducing the risk of misclassification bias. To the best of our knowledge, this is the first study to analyze the linear relationships between WC and fracture risk at skeletal sites other than the hip and vertebrae and to compare both BMI and WC exposures in the same cohort of middle‐aged individuals. Finally, we conducted multiple sensitivity analyses to assess the robustness of our results.

Our study also has several limitations. First, our cohort was composed of middle‐aged adults among which fractures do not occur as often as in the elderly, reducing our statistical power to find associations at skeletal sites with a low incidence of fracture events. Second, although 10% of individuals had type 2 diabetes at recruitment, a limited number of incident fractures were found in this group, which did not provide sufficient power for the analysis of interaction. Third, potentially confounding factors, such as history of falls and markers of mineral metabolism (serum calcium, 25‐hydroxyvitamin D, and parathyroid hormone), were not available in the cohort. Nonetheless, we had data on calcium and vitamin D supplementation, and a previous study had found normal levels of calcium, phosphate, and parathyroid hormone in a subgroup of the CARTaGENE cohort.^(^ ^45^ ^)^ Furthermore, it was not possible to know whether exposures or confounding factors changed during follow‐up since all variables were measured once at recruitment. Lastly, because the majority of our cohort was of European descent, results may not be generalizable to other ethnic groups.

Finally, BMD by dual‐energy X‐ray absorptiometry (DXA) was not measured in the CARTaGENE cohort. Thus, results of our analysis are not controlled for BMD. However, based on the literature and our DAG, we considered that BMD may fall somewhere in the causal chain between obesity and fractures. Indeed, numerous studies found a strong positive association between BMI and BMD, showing that a higher BMI was positively associated with a higher BMD, mainly because of greater weight.^(^ ^46^, ^47^, ^48^, ^49^ ^)^ Then, adjusting for BMD would block an indirect pathway between BMI and fracture incidence that is part of the association, resulting in a total effect that is underestimated. Moreover, models are already adjusted for multiple factors that are strong predictors of both fractures and BMD, such as age, sex, menopausal status, osteoporosis diagnosis and medication, history of fractures, and use of glucocorticoids, that are not in the causal pathways between obesity and fractures.

In conclusion, middle‐aged individuals with obesity, and particularly those with abdominal obesity, are at a higher risk of incident distal lower limb and ankle fractures. This has major public health implications since individuals with obesity who sustain a fracture are more likely to have comorbidities that may cause slower rehabilitation, greater risk of nonunion, and postoperative complications,^(^ ^50^, ^51^, ^52^ ^)^ generating substantial healthcare costs. The predicted increase in fracture incidence associated with the aging of the population and the rising incidence of obesity may exacerbate the fracture burden over the coming years. Understanding the mechanisms by which adults with obesity are more susceptible to certain fracture sites is necessary to develop effective prevention strategies.

## AUTHOR CONTRIBUTIONS

**Anne‐Frédérique Turcotte:** Conceptualization; data curation; formal analysis; methodology; writing – original draft; writing – review and editing. **Sonia Jean:** Conceptualization; methodology; supervision; writing – review and editing. **Suzanne Nicole Morin:** Writing – review and editing. **Fabrice Mac‐Way:** Data curation; writing – review and editing. **Claudia Gagnon:** Conceptualization; methodology; supervision; writing – review and editing.

### PEER REVIEW

The peer review history for this article is available at https://publons.com/publon/10.1002/jbm4.10730.

## Supporting information


**Appendix S1.** Supporting information.
