# Supplementary material for: Relationships between Obesity and Incidence of Fractures in a Middle‐Aged Population: A Study from the CARTaGENE Cohort
Source: JBMR Plus. 2023 Mar 3;7(5):e10730. doi: 10.1002/jbm4.10730 (PMC10184011; doi:10.1002/jbm4.10730)
Supplement: Supplementary file 1 — Appendix S1. Supporting information. [file JBM4-7-e10730-s001.docx]

**Supplementary Table 1**. Associations between waist circumference (in category) and incidence of fractures for the whole cohort.

| **Fracture outcome** | **Waist circumference^a^** | **Incident fracture, n** | **Crude HR (95% CI)** | **P value** | **Adjusted^b^ HR (95% CI)** | **P value** |
| --- | --- | --- | --- | --- | --- | --- |
| Any fracture | <80 or <94 cm | 235 | Reference |  | Reference |  |
|  | 80-87.9 or 94-101.9 cm | 184 | 0.99 (0.82, 1.20) | 0.935 | 0.96 (0.79, 1.18) | 0.709 |
|  | ≥88 or ≥102 cm | 401 | 1.27 (1.08, 1.49) | 0.004 | 1.15 (0.96, 1.36) | 0.124 |
|  |  |  |  |  |  |  |
| MOF | <80 or <94 cm | 131 | Reference |  | Reference |  |
|  | 80-87.9 or 94-101.9 cm | 83 | 0.80 (0.61, 1.06) | 0.118 | 0.76 (0.58, 1.01) | 0.062 |
|  | ≥88 or ≥102 cm | 201 | 1.14 (0.92, 1.42) | 0.239 | 0.98 (0.77, 1.24) | 0.833 |
|  |  |  |  |  |  |  |
| Distal lower limb | <80 or <94 cm | 78 | Reference |  | Reference |  |
|  | 80-87.9 or 94-101.9 cm | 89 | 1.45 (1.07, 1.96) | 0.018 | 1.39 (1.06, 1.90) | 0.040 |
|  | ≥88 or ≥102 cm | 186 | 1.77 (1.36, 2.31) | <0.001 | 1.61 (1.22, 2.13) | 0.001 |
|  |  |  |  |  |  |  |
| Distal upper limb | <80 or <94 cm | 54 | Reference |  | Reference |  |
|  | 80-87.9 or 94-101.9 cm | 44 | 1.03 (0.69, 1.54) | 0.875 | 1.05 (0.70, 1.58) | 0.808 |
|  | ≥88 or ≥102 cm | 105 | 1.45 (1.04, 2.01) | 0.028 | 1.37 (0.96, 1.94) | 0.080 |
|  |  |  |  |  |  |  |

^a^Cut-off points for women: <80 cm; 80-87.9 cm; ≥88. Cut-off points for men: <94 cm; 94-101.9 cm; ≥102 cm. Ethic-specific cut-offs have been used.

^b^Model adjusted for age, sex, menopausal status, area of residence, ethnicity, education, annual income, marital status, smoking, alcohol intake, physical activity level, calcium and vitamin D supplementation, history of fracture, osteoporosis, hyperthyroidism, chronic kidney disease, type 1 diabetes, liver disease, chronic obstructive pulmonary disease, cardiovascular disease, cancer, osteoporosis drugs, glucocorticoids, anticonvulsants, oral contraceptive, hormonal replacement therapy, proton pump inhibitor, blood pressure medication.

**Supplementary Table 2**. Associations between body mass index (in category) and incidence of fractures for the whole cohort.

| **Fracture outcome** | **Body mass index** | **Incident fracture, n** | **Crude HR (95% CI)** | **P value** | **Adjusted^a^ HR (95% CI)** | **P value** |
| --- | --- | --- | --- | --- | --- | --- |
| Any fracture | <18.5 | 11 | 1.71 (0.94, 3.12) | 0.080 | 1.33 (0.73, 2.46) | 0.354 |
|  | 18.5-24.9 | 252 | 1.02 (0.86, 1.20) | 0.853 | 0.95 (0.79, 1.13) | 0.543 |
|  | 25.0-29.9 | 296 | Reference |  | Reference |  |
|  | ≥30 | 217 | 1.08 (0.90, 1.28) | 0.414 | 1.03 (0.85, 1.23) | 0.786 |
|  |  |  |  |  |  |  |
| MOF | <18.5 | 8 | 2.71 (1.33, 5.52) | 0.006 | 2.04 (0.99, 4.22) | 0.054 |
|  | 18.5-24.9 | 138 | 1.21 (0.96, 1.53) | 0.114 | 1.14 (0.89, 1.46) | 0.297 |
|  | 25.0-29.9 | 136 | Reference |  | Reference |  |
|  | ≥30 | 110 | 1.19 (0.92, 1.53) | 0.182 | 1.12 (0.86, 1.46) | 0.387 |
|  |  |  |  |  |  |  |
| Distal lower limb | <18.5 | 2 | 0.66 (0.16, 2.66) | 0.558 | 0.50 (0.12, 2.05) | 0.337 |
|  | 18.5-24.9 | 94 | 0.80 (0.62, 1.04) | 0.097 | 0.72 (0.55, 0.96) | 0.023 |
|  | 25.0-29.9 | 140 | Reference |  | Reference |  |
|  | ≥30 | 100 | 1.05 (0.81, 1.36) | 0.719 | 0.98 (0.75, 1.29) | 0.900 |
|  |  |  |  |  |  |  |
| Distal upper limb | <18.5 | 3 | 1.89 (0.60, 6.01) | 0.279 | 1.30 (0.40, 4.18) | 0.660 |
|  | 18.5-24.9 | 68 | 1.11 (0.80, 1.55) | 0.530 | 0.97 (0.69, 1.37) | 0.873 |
|  | 25.0-29.9 | 73 | Reference |  | Reference |  |
|  | ≥30 | 58 | 1.17 (0.83, 1.65) | 0.383 | 1.15 (0.80, 1.65) | 0.444 |
|  |  |  |  |  |  |  |

^a^Model adjusted for age, sex, menopausal status, area of residence, ethnicity, education, annual income, marital status, smoking, alcohol intake, physical activity level, calcium and vitamin D supplementation, history of fracture, osteoporosis, hyperthyroidism, chronic kidney disease, type 1 diabetes, liver disease, chronic obstructive pulmonary disease, cardiovascular disease, cancer, osteoporosis drugs, glucocorticoids, anticonvulsants, oral contraceptive, hormonal replacement therapy, proton pump inhibitor, blood pressure medication.

**Supplementary Table 3**. Results of sensitivity analyses: competitive risk of mortality, exclusion of individuals with a diagnosis of osteoporosis or taking an osteoporosis medication, and exclusion of individuals aged 40-49 years.

| **Competitive risk of mortality** | | | **Waist circumference** | | | | | **Body mass index** | | |
| --- | --- | --- | --- | --- | --- | --- | --- | --- | --- | --- |
| **Fracture outcome** | | **Group** | | **Incident fracture, n** | **Deaths, n** | **Adjusted HR per 10-cm increase (95% CI)** | **P value** | **Incident fracture, n** | **Deaths, n** | **Adjusted P value for spline** |
| Any fracture | Whole cohort | | | 775 | 199 | 1.04 (0.98, 1.10) | 0.193 | 736 | 176 | 0.419 |
|  | Women | | | 473 | 95 | 1.05 (0.98, 1.12) | 0.190 | 453 | 90 | 0.690 |
|  | Men | | | 302 | 104 | 1.01 (0.92, 1.11) | 0.863 | 283 | 86 | 0.040 |
| MOF | Whole cohort | | | 394 | 205 | 0.99 (0.91, 1.08) | 0.867 | 376 | 182 | 0.118 |
|  | Women | | | 248 | 96 | 1.00 (0.90, 1.11) | 0.991 | 242 | 91 | 0.181 |
|  | Men | | | 146 | 109 | 0.98 (0.85, 1.13) | 0.742 | 134 | 91 | 0.042 |
| Distal lower limb | Whole cohort | | | 331 | 212 | 1.12 (1.04, 1.20) | 0.003 | 314 | 189 | 0.018 |
|  | Women | | | 207 | 101 | 1.12 (1.02, 1.23) | 0.019 | 197 | 96 | 0.056 |
|  | Men | | | 124 | 111 | 1.11 (0.99, 1.25) | 0.085 | 117 | 93 | 0.521 |
| Distal upper limb | Whole cohort | | | 195 | 214 | 1.08 (0.97, 1.21) | 0.154 | 194 | 191 | 0.333 |
|  | Women | | | 137 | 101 | 1.09 (0.96, 1.25) | 0.180 | 135 | 96 | 0.181 |
|  | Men | | | 58 | 113 | 1.03 (0.83, 1.27) | 0.814 | 59 | 95 | 0.037 |
| **Exclusion of individuals with a diagnosis of osteoporosis and taking an osteoporosis medication** | | | | | | | | | | |
| Any fracture | Whole cohort | | | 664 |  | 1.06 (1.00, 1.12) | 0.062 | 631 |  | 0.595 |
|  | Women | | | 374 |  | 1.08 (1.00, 1.16) | 0.041 | 359 |  | 0.858 |
|  | Men | | | 290 |  | 1.01 (0.92, 1.11) | 0.879 | 272 |  | 0.140 |
| MOF | Whole cohort | | | 326 |  | 1.01 (0.93, 1.10) | 0.763 | 310 |  | 0.052 |
|  | Women | | | 187 |  | 1.02 (0.92, 1.14) | 0.665 | 183 |  | 0.131 |
|  | Men | | | 139 |  | 0.98 (0.85, 1.13) | 0.776 | 127 |  | 0.083 |
| Distal lower limb | Whole cohort | | | 291 |  | 1.14 (1.05, 1.24) | 0.002 | 277 |  | 0.013 |
|  | Women | | | 172 |  | 1.16 (1.04, 1.28) | 0.007 | 164 |  | 0.025 |
|  | Men | | | 119 |  | 1.11 (0.97, 1.28) | 0.139 | 113 |  | 0.519 |
| Distal upper limb | Whole cohort | | | 165 |  | 1.09 (0.97, 1.22) | 0.160 | 165 |  | 0.373 |
|  | Women | | | 108 |  | 1.11 (0.96, 1.27) | 0.163 | 108 |  | 0.384 |
|  | Men | | | 57 |  | 1.02 (0.83, 1.26) | 0.848 | 57 |  | 0.036 |
| **Exclusion of individuals aged 40-49 years** | | | | | | | | | | |
| Any fracture | Whole cohort | | | 584 |  | 1.05 (0.98, 1.11) | 0.159 | 551 |  | 0.394 |
|  | Women | | | 380 |  | 1.06 (0.99, 1.15) | 0.109 | 360 |  | 0.486 |
|  | Men | | | 204 |  | 1.00 (0.89, 1.12) | 0.984 | 191 |  | 0.181 |
| MOF | Whole cohort | | | 320 |  | 0.99 (0.91, 1.08) | 0.873 | 302 |  | 0.411 |
|  | Women | | | 211 |  | 1.03 (0.93, 1.14) | 0.605 | 204 |  | 0.610 |
|  | Men | | | 109 |  | 0.91 (0.78, 1.07) | 0.268 | 98 |  | 0.307 |
| Distal lower limb | Whole cohort | | | 231 |  | 1.16 (1.06, 1.28) | 0.002 | 218 |  | 0.021 |
|  | Women | | | 155 |  | 1.14 (1.01, 1.27) | 0.029 | 145 |  | 0.062 |
|  | Men | | | 76 |  | 1.22 (1.02, 1.45) | 0.028 | 73 |  | 0.341 |
| Distal upper limb | Whole cohort | | | 159 |  | 1.10 (0.98, 1.24) | 0.104 | 156 |  | 0.121 |
|  | Women | | | 118 |  | 1.13 (0.98, 1.29) | 0.086 | 115 |  | 0.122 |
|  | Men | | | 41 |  | 1.02 (0.79, 1.32) | 0.676 | 41 |  | 0.065 |

HR: hazard ratio; 95% CI: 95% confidence intervals.

**Supplementary Table 4**. Linear relationships between waist circumference and incidence of humerus and ankle fractures.

| **Fracture outcome** | **Group** | **Waist circumference (cm)** | **Incident fracture, n** | **Crude HR (95% CI)** | **P value** | **Adjusted HR (95% CI)** | **P value** |
| --- | --- | --- | --- | --- | --- | --- | --- |
| Humerus | Whole cohort | HR per 10-cm increase | 103 | 0.99 (0.87, 1.13) | 0.886 | 1.02 (0.87, 1.18) | 0.830 |
|  |  |  |  |  |  |  |  |
|  | Women | HR per 10-cm increase | 66 | 1.07 (0.90, 1.26) | 0.441 | 1.00 (0.83, 1.20) | 0.994 |
|  |  |  |  |  |  |  |  |
|  | Men | HR per 10-cm increase | 37 | 1.05 (0.83, 1.34) | 0.675 | 1.08 (0.83, 1.41) | 0.546 |
| Ankle | Whole cohort | HR per 10-cm increase | 127 | 1.13 (1.01, 1.27) | 0.034 | 1.21 (1.07, 1.37) | 0.003 |
|  |  |  |  |  |  |  |  |
|  | Women | HR per 10-cm increase | 80 | 1.20 (1.04, 1.38) | 0.012 | 1.19 (1.02, 1.39) | 0.026 |
|  |  |  |  |  |  |  |  |
|  | Men | HR per 10-cm increase | 47 | 1.24 (1.02, 1.51) | 0.031 | 1.25 (1.01, 1.56) | 0.044 |

HR: hazard ratio; 95% CI: 95% confidence intervals.

**Supplementary Table 5**. Non-linear relationships between body mass index and incidence of humerus and ankle fractures.

| **Fracture outcome** | **Group** | **Incident fracture, n** | **Crude P value for spline** | **Adjusted P value for spline** |
| --- | --- | --- | --- | --- |
| Humerus | Whole cohort | 92 | 0.101 | 0.357 |
|  | Women | 65 | 0.195 | 0.165 |
|  | Men | 27 | 0.354 | 0.145 |
| Ankle | Whole cohort | 122 | 0.048 | 0.026 |
|  | Women | 78 | 0.099 | 0.092 |
|  | Men | 44 | 0.191 | 0.442 |

**Supplementary Figure 1**. Directed acyclic graph.


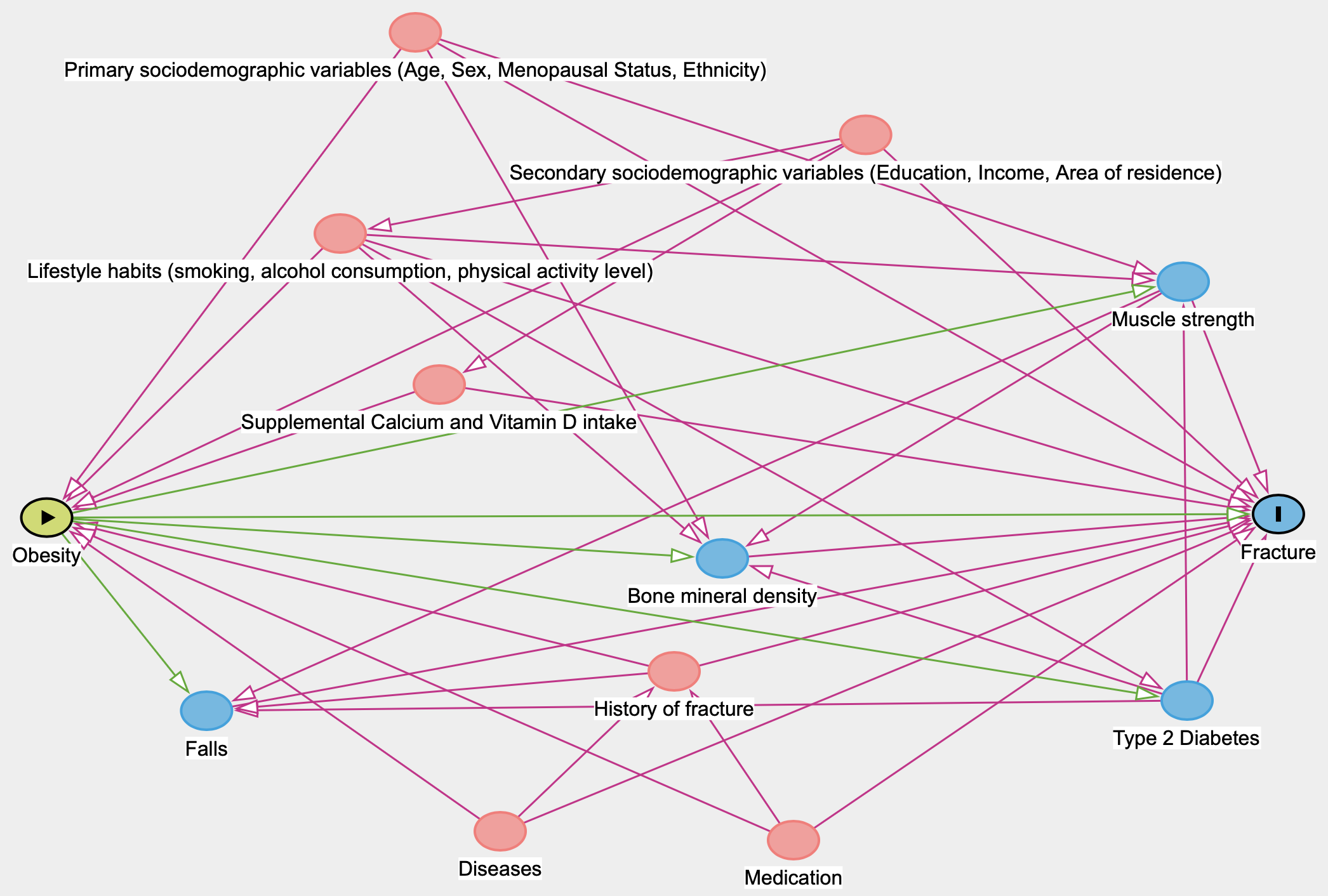


Diseases: osteoporosis, hyperthyroidism, type 1 diabetes, chronic kidney disease, liver disease, chronic obstructive pulmonary disease, cardiovascular disease, cancer.

Medication: osteoporosis drugs, glucocorticoids, anticonvulsants, oral contraceptive, hormonal replacement therapy, proton pump inhibitor, blood pressure medication.

**Supplementary Figure 2**. Relationships between A) waist circumference and B) body mass index, and incidence of ankle fractures in the whole cohort and in men and women separately. HRs are per 10 cm increase in waist circumference.


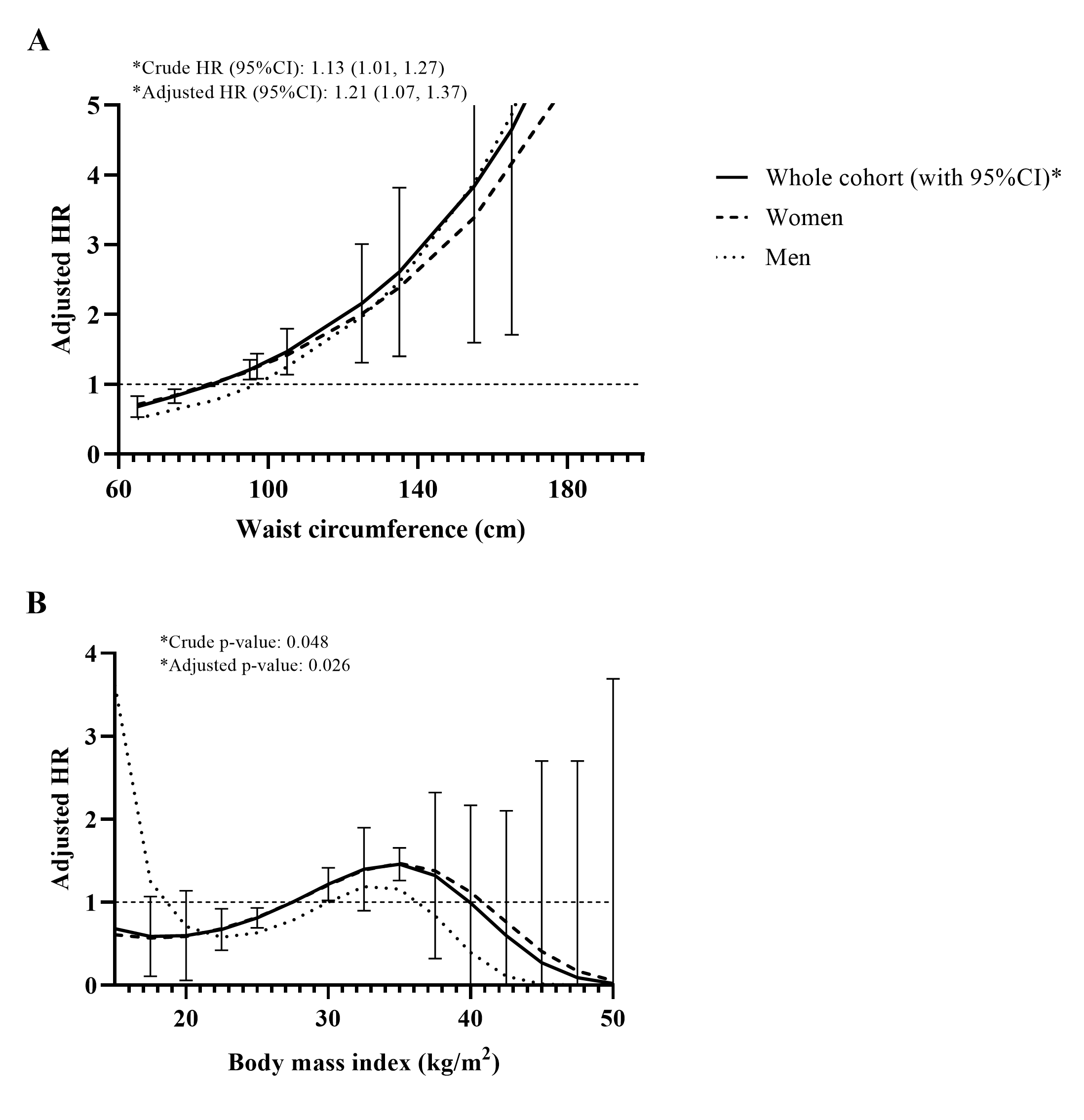


HR: hazard ratio; 95%CI: 95% confidence interval.

Models are adjusted for: age, sex, menopausal status, area of residence, ethnicity, education, annual income, marital status, smoking, alcohol intake, physical activity level, calcium and vitamin D supplementation, history of fracture, osteoporosis, hyperthyroidism, chronic kidney disease, type 1 diabetes, liver disease, chronic obstructive pulmonary disease, cardiovascular disease, cancer, osteoporosis drugs, glucocorticoids, anticonvulsants, oral contraceptive, hormonal replacement therapy, proton pump inhibitor, blood pressure medication.
